# Crystal structure of tetra­kis­(μ-*N*-phenyl­acetamidato)-κ^4^ *N*:*O*;κ^4^ *O*:*N*-bis­[(2-methyl­benzo­nitrile-κ*N*)rhodium(II)](*Rh*—*Rh*)

**DOI:** 10.1107/S1600536814017930

**Published:** 2014-08-20

**Authors:** Cassandra T. Eagle, Nkongho Atem-Tambe, Kenneth K. Kpogo, Jennie Tan, Kevin M. Cook

**Affiliations:** a Department of Chemistry, East Tennessee State University, PO Box 70695, Johnson City, TN 37614, USA

**Keywords:** crystal structure, Rh^II^ complex, dirhodium core, acetamidate ligand

## Abstract

The complex molecule of the title compound, [Rh_2_{N(C_6_H_5_)COCH_3_}_4_(C_8_H_7_N)_2_], exhibits inversion symmetry. The four acetamidate ligands bridging the dirhodium core are arranged in a 2,2-*trans* manner with two N atoms and two O atoms coordinating to each Rh^II^ atom *trans* to one another. The N_eq_—Rh—Rh—O_eq_ torsion angles on the acetamidate bridge vary between −4.07 (5) and −6.78 (7)°. The axial nitrile ligands complete the distorted octa­hedral coordination sphere of each Rh^II^ atom and show a nonlinear coordination with Rh—N—C bond angles of 151.6 (3) and 152.5 (3)°. The bond lengths of the two nitrile triple bonds are 1.133 (5) and 1.137 (5) Å.

## Related literature   

For the synthesis and structures of four related compounds, see: Lifsey *et al.* (1987 ▶); Eagle *et al.* (2000 ▶, 2012 ▶, 2013*a* ▶,*b* ▶, 2014 ▶).
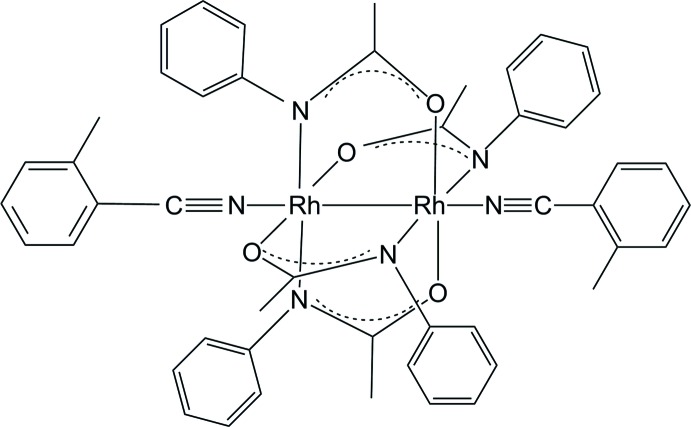


## Experimental   

### Crystal data  

[Rh_2_(C_8_H_8_NO)_4_(C_8_H_7_N)_2_]
*M* *_r_* = 976.74
Triclinic, 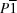

*a* = 9.7912 (7) Å
*b* = 14.7873 (10) Å
*c* = 16.3592 (11) Å
α = 103.837 (7)°
β = 99.173 (7)°
γ = 99.772 (7)°
*V* = 2216.4 (3) Å^3^
*Z* = 2
Mo *K*α radiation
μ = 0.79 mm^−1^
*T* = 223 K
0.33 × 0.12 × 0.12 mm

### Data collection  

Rigaku XtaLAB mini diffractometer
Absorption correction: multi-scan (*REQAB*; Rigaku, 1998 ▶) *T*_min_ = 0.720, *T*_max_ = 0.909
23557 measured reflections
10137 independent reflections
7966 reflections with *F* ^2^ > 2σ(*F* ^2^)
*R*_int_ = 0.039

### Refinement  

*R*[*F* ^2^ > 2σ(*F* ^2^)] = 0.037
*wR*(*F* ^2^) = 0.079
*S* = 1.04
10137 reflections
547 parameters
H-atom parameters constrained
Δρ_max_ = 0.62 e Å^−3^
Δρ_min_ = −0.51 e Å^−3^

### 

Data collection: *CrystalClear* (Rigaku, 2011 ▶); cell refinement: *CrystalClear*; data reduction: *CrystalClear*; program(s) used to solve structure: *SIR92* (Altomare, *et al.*, 1994 ▶); program(s) used to refine structure: *SHELXL97* (Sheldrick, 2008 ▶); molecular graphics: *CrystalStructure* (Rigaku, 2011 ▶); software used to prepare material for publication: *CrystalStructure*.

## Supplementary Material

Crystal structure: contains datablock(s) General, I. DOI: 10.1107/S1600536814017930/wm5039sup1.cif

Structure factors: contains datablock(s) I. DOI: 10.1107/S1600536814017930/wm5039Isup2.hkl

**Fig. 1.**
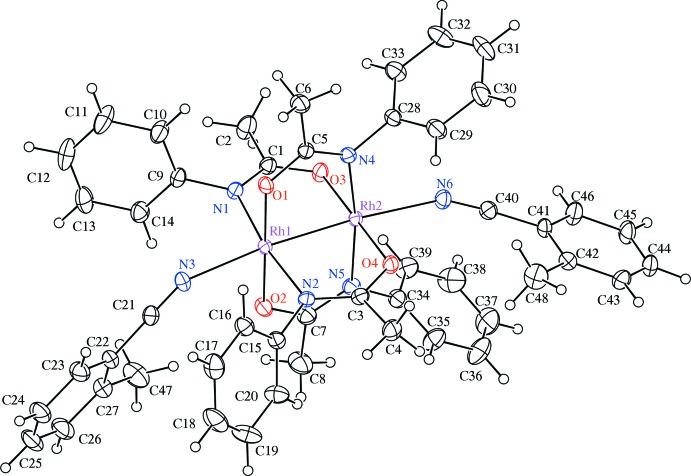
The molecular structure of the title compound with displacement ellipsoids at the 30% probability level. Hydrogen atoms are drawn as small spheres. The numbering scheme of the title compound is adopted from a related compound (Eagle et al., 2000).

Supporting information file. DOI: 10.1107/S1600536814017930/wm5039Isup3.txt

et al. . DOI: 10.1107/S1600536814017930/wm5039fig1.tif
The mol­ecular structure of the title compound with displacement ellipsoids at the 30% probability level. Hydrogen atoms are drawn as small spheres. The numbering scheme of the title compound is adopted from a related compound (Eagle *et al.*, 2000).

**Fig. 2.**
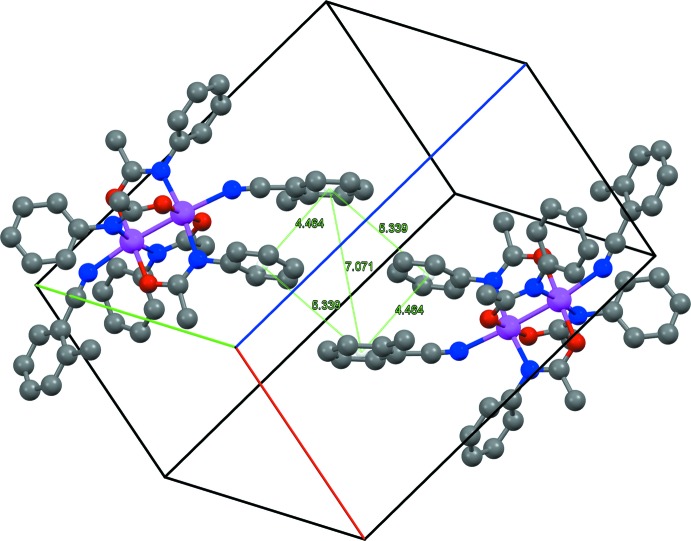
The packing diagram for the title compound.

. DOI: 10.1107/S1600536814017930/wm5039fig2.tif
The packing diagram for the title compound.

CCDC reference: 1017877

Additional supporting information:  crystallographic information; 3D view; checkCIF report

## Figures and Tables

**Table:** 

Rh1—Rh2	2.4241 (4)
Rh1—O1	2.034 (2)
Rh1—O2	2.028 (3)
Rh1—N1	2.061 (2)
Rh1—N2	2.071 (2)
Rh1—N3	2.236 (3)
Rh2—O3	2.0358 (17)
Rh2—O4	2.0279 (17)
Rh2—N4	2.048 (3)
Rh2—N5	2.067 (3)
Rh2—N6	2.254 (3)

**Table:** 

Rh2—Rh1—N3	172.79 (7)
Rh1—Rh2—N6	174.59 (6)

## supplementary crystallographic information

### S1. Synthesis and crystallization

Approximately 20 mg of *trans*-tetra­kis[µ-N-(phenyl)­acetamidato]-κ^4^N:O;κ^4^O:N rhodium(II) was dissolved in 5 ml di­chloro­methane. 6.4 µl of 2-methyl benzo­nitrile was then added to this solution, via a gas tight syringe, turning the solution from a green to a dark blue color. The blue solution then turned red over time. Crystals grew over a two week period via vapor diffusion. From the structure determination, the title compound is an adduct of *trans*-tetra­kis[µ-N-(phenyl)­acetamidato]-κ^4^N:O;κ^4^O:N rhodium(II) with 2-methyl benzo­nitrile in each axial site of the Rh—Rh dumbbell.

### S2. Refinement

Crystal data, data collection and structure refinement details are summarized in Table 1. H atoms were placed in geometrically idealized positions and constrained to ride on their parent atoms with a C—H distance of 0.93 (aromatic) and *U*_iso_(H) = 1.2*U*_eq_(C), and with 0.98 Å (methyl) and *U*_iso_(H)= 1.5*U*_eq_(C).

Thirteen reflections were omitted from the refinement due to strong differences between observed and calculated intensities. They may have been low-angle reflections obscured from the beamstop, or the relections may have overloaded in the detector (even in the overload-correction mode).

### Crystal data

**Table:**

[Rh_2_(C_8_H_8_NO)_4_(C_8_H_7_N)_2_]	*Z* = 2
*M**_r_* = 976.74	*F*(000) = 996.00
Triclinic, *P*1	*D*_x_ = 1.463 Mg m^−^^3^
Hall symbol: -P 1	Mo *K*α radiation, λ = 0.71075 Å
*a* = 9.7912 (7) Å	Cell parameters from 19839 reflections
*b* = 14.7873 (10) Å	θ = 3.0–27.6°
*c* = 16.3592 (11) Å	µ = 0.79 mm^−^^1^
α = 103.837 (7)°	*T* = 223 K
β = 99.173 (7)°	Block, red
γ = 99.772 (7)°	0.33 × 0.12 × 0.12 mm
*V* = 2216.4 (3) Å^3^	

### Data collection

**Table:**

Rigaku XtaLAB mini diffractometer	10137 independent reflections
Radiation source: fine-focus sealed X-ray tube	7966 reflections with *F*^2^ > 2σ(*F*^2^)
Graphite Monochromator monochromator	*R*_int_ = 0.039
Detector resolution: 6.827 pixels mm^-1^	θ_max_ = 27.5°
ω scans	*h* = −12→12
Absorption correction: multi-scan (*REQAB*; Rigaku, 1998)	*k* = −19→19
*T*_min_ = 0.720, *T*_max_ = 0.909	*l* = −21→21
23557 measured reflections	

### Refinement

**Table:**

Refinement on *F*^2^	Primary atom site location: structure-invariant direct methods
Least-squares matrix: full	Secondary atom site location: difference Fourier map
*R*[*F*^2^ > 2σ(*F*^2^)] = 0.037	Hydrogen site location: inferred from neighbouring sites
*wR*(*F*^2^) = 0.079	H-atom parameters constrained
*S* = 1.04	*w* = 1/[σ^2^(*F*_o_^2^) + (0.0257*P*)^2^ + 1.5457*P*] where *P* = (*F*_o_^2^ + 2*F*_c_^2^)/3
10137 reflections	(Δ/σ)_max_ = 0.002
547 parameters	Δρ_max_ = 0.62 e Å^−^^3^
0 restraints	Δρ_min_ = −0.51 e Å^−^^3^
0 constraints	

### Special details

**Table:**

Experimental. The infrared absorption spectum of the title compound showed a band at 2320 cm^-1^ attributable to C—N bond stretching modes. The corresponding band for uncomplexed 2-methyl benzonitrile appears at 2224 cm^-1^. This indicates that there is a shortening of the C—N bond and a stronger σ-interaction to the rhodium metal compared to the π-back bonding which occurs upon complexation with *trans-*tetrakis[µ-N-(phenyl)acetamidato]-κ^4^N:O;κ^4^O:N rhodium(II). The predominance of σ-bonding in the Rh—N—C bond system is consistent with the lack of the linear bond angles that would be expected if π-back bonding was more prevalent.
Geometry. Compound 1 has the methyl group of the 2-methyl benzonitrile ligand pointing into empty space because the methyl group is more bulky than the hydrogen atoms and will overlap with the phenyl rings on the phenylacetamide bridge. From the packing diagram (see Fig 2), it can be seen that both nitrile groups on the 2-methyl benzonitrile ligands are bent in the same direction. The predominance of σ-bonding in the rhodium-nitrogen-carbon bond system (and lower affect of π-back bonding) is the likely cause of this deviation from linearity, though an argument can be be made for packing forces to account for the severity of this deviation. For example, in compound 2 (Eagle *et al.*, 2000) there are no methyl groups on the benzonitrile ligand nor on the phenyl rings of the phenyl acetamide bridge and the rhodium-nitrogen-carbon bond angles are closer to linear. The packing diagram shows that an acetamide phenyl ring and the tolunitrile ligand on the same rhodium are stacked upon each other.
Refinement. Refinement was performed using all reflections. The weighted *R*-factor (*wR*) and goodness of fit (*S*) are based on *F*^2^. *R*-factor (gt) are based on *F*. The threshold expression of *F*^2^ > 2.0 σ(*F*^2^) is used only for calculating *R*-factor (gt).

### Fractional atomic coordinates and isotropic or equivalent isotropic displacement parameters (Å^2^)

**Table:**

	*x*	*y*	*z*	*U*_iso_*/*U*_eq_	
Rh1	1.01287 (2)	0.209087 (15)	0.807834 (13)	0.02313 (6)	
Rh2	0.99761 (2)	0.274621 (15)	0.685109 (13)	0.02373 (6)	
O1	1.1252 (2)	0.11655 (13)	0.75245 (12)	0.0292 (5)	
O2	0.9034 (3)	0.30262 (14)	0.86384 (13)	0.0351 (5)	
O3	1.1707 (2)	0.37983 (13)	0.74879 (13)	0.0314 (5)	
O4	0.8259 (2)	0.16918 (14)	0.62181 (12)	0.0320 (5)	
N1	1.2007 (3)	0.30885 (16)	0.85771 (15)	0.0281 (5)	
N2	0.8227 (3)	0.11663 (16)	0.74200 (15)	0.0276 (5)	
N3	1.0082 (3)	0.16001 (17)	0.92666 (15)	0.0317 (6)	
N4	1.1241 (3)	0.18475 (16)	0.64161 (15)	0.0273 (5)	
N5	0.8720 (3)	0.35709 (17)	0.74472 (16)	0.0318 (6)	
N6	0.9624 (3)	0.32712 (17)	0.56656 (16)	0.0340 (6)	
C1	1.2406 (3)	0.3749 (2)	0.82002 (19)	0.0304 (7)	
C2	1.3725 (4)	0.4523 (3)	0.8583 (3)	0.0488 (9)	
C3	0.7702 (3)	0.1132 (2)	0.66219 (19)	0.0305 (7)	
C4	0.6393 (4)	0.0417 (3)	0.6085 (2)	0.0442 (8)	
C5	1.1626 (3)	0.1240 (2)	0.68232 (18)	0.0280 (6)	
C6	1.2568 (4)	0.0586 (3)	0.6509 (2)	0.0408 (8)	
C7	0.8534 (4)	0.3571 (2)	0.8218 (2)	0.0350 (7)	
C8	0.7708 (5)	0.4210 (3)	0.8705 (3)	0.0575 (11)	
C9	1.2896 (3)	0.30217 (19)	0.93267 (18)	0.0300 (7)	
C10	1.4003 (4)	0.2568 (3)	0.9263 (3)	0.0471 (9)	
C11	1.4856 (5)	0.2479 (3)	0.9991 (3)	0.0612 (11)	
C12	1.4571 (5)	0.2835 (3)	1.0785 (3)	0.0595 (11)	
C13	1.3473 (5)	0.3290 (3)	1.0857 (3)	0.0528 (10)	
C14	1.2626 (4)	0.3381 (3)	1.0133 (2)	0.0411 (8)	
C15	0.7454 (3)	0.0593 (2)	0.78493 (18)	0.0306 (7)	
C16	0.7892 (4)	−0.0200 (2)	0.8014 (2)	0.0371 (7)	
C17	0.7152 (4)	−0.0741 (3)	0.8457 (3)	0.0509 (9)	
C18	0.5991 (5)	−0.0483 (3)	0.8741 (3)	0.0610 (11)	
C19	0.5560 (4)	0.0306 (3)	0.8590 (3)	0.0625 (12)	
C20	0.6296 (4)	0.0852 (3)	0.8156 (3)	0.0453 (9)	
C21	0.9643 (3)	0.1564 (2)	0.98608 (19)	0.0320 (7)	
C22	0.9072 (4)	0.1529 (3)	1.06197 (19)	0.0344 (7)	
C23	0.8885 (4)	0.0686 (3)	1.0857 (3)	0.0456 (9)	
C24	0.8369 (4)	0.0646 (3)	1.1588 (3)	0.0556 (10)	
C25	0.8049 (5)	0.1447 (4)	1.2067 (3)	0.0617 (11)	
C26	0.8222 (4)	0.2281 (3)	1.1829 (3)	0.0548 (10)	
C27	0.8739 (4)	0.2347 (3)	1.1099 (2)	0.0422 (8)	
C28	1.1614 (3)	0.1857 (2)	0.56093 (18)	0.0296 (7)	
C29	1.0725 (4)	0.1283 (3)	0.4856 (2)	0.0465 (9)	
C30	1.1042 (5)	0.1310 (3)	0.4068 (3)	0.0600 (11)	
C31	1.2234 (5)	0.1921 (3)	0.4025 (3)	0.0569 (11)	
C32	1.3119 (5)	0.2491 (3)	0.4771 (3)	0.0540 (10)	
C33	1.2809 (4)	0.2465 (3)	0.5562 (2)	0.0417 (8)	
C34	0.8123 (4)	0.4202 (2)	0.7027 (2)	0.0350 (7)	
C35	0.6725 (4)	0.3946 (3)	0.6589 (3)	0.0496 (9)	
C36	0.6154 (5)	0.4555 (3)	0.6174 (3)	0.0661 (12)	
C37	0.6983 (5)	0.5406 (3)	0.6186 (3)	0.0676 (12)	
C38	0.8372 (5)	0.5652 (3)	0.6605 (3)	0.0633 (12)	
C39	0.8955 (4)	0.5051 (3)	0.7022 (3)	0.0457 (9)	
C40	0.8918 (4)	0.3379 (2)	0.5090 (2)	0.0332 (7)	
C41	0.7953 (3)	0.3515 (2)	0.43923 (18)	0.0318 (7)	
C42	0.6750 (3)	0.2806 (2)	0.39791 (19)	0.0335 (7)	
C43	0.5801 (4)	0.2994 (3)	0.3338 (2)	0.0423 (8)	
C44	0.6058 (4)	0.3844 (3)	0.3127 (2)	0.0438 (8)	
C45	0.7254 (4)	0.4533 (3)	0.3527 (2)	0.0440 (8)	
C46	0.8207 (4)	0.4368 (3)	0.4165 (2)	0.0438 (8)	
C47	0.8930 (5)	0.3266 (3)	1.0843 (3)	0.0604 (11)	
C48	0.6476 (4)	0.1880 (3)	0.4222 (3)	0.0549 (10)	
H2A	1.3468	0.5130	0.8742	0.0586*	
H2B	1.4314	0.4529	0.8167	0.0586*	
H2C	1.4235	0.4403	0.9084	0.0586*	
H4A	0.6637	0.0006	0.5606	0.0530*	
H4B	0.5707	0.0746	0.5877	0.0530*	
H4C	0.6000	0.0042	0.6431	0.0530*	
H6A	1.2281	−0.0017	0.6624	0.0489*	
H6B	1.3533	0.0867	0.6802	0.0489*	
H6C	1.2492	0.0490	0.5901	0.0489*	
H8A	0.8244	0.4514	0.9280	0.0691*	
H8B	0.6820	0.3835	0.8727	0.0691*	
H8C	0.7538	0.4688	0.8417	0.0691*	
H10	1.4185	0.2315	0.8724	0.0565*	
H11	1.5614	0.2182	0.9941	0.0734*	
H12	1.5125	0.2767	1.1275	0.0714*	
H13	1.3296	0.3541	1.1398	0.0634*	
H14	1.1874	0.3684	1.0188	0.0493*	
H16	0.8684	−0.0373	0.7828	0.0445*	
H17	0.7445	−0.1277	0.8561	0.0611*	
H18	0.5497	−0.0845	0.9037	0.0732*	
H19	0.4769	0.0477	0.8780	0.0750*	
H20	0.6011	0.1397	0.8068	0.0543*	
H23	0.9107	0.0148	1.0525	0.0547*	
H24	0.8241	0.0083	1.1753	0.0668*	
H25	0.7708	0.1425	1.2562	0.0740*	
H26	0.7988	0.2812	1.2164	0.0658*	
H29	0.9906	0.0875	0.4879	0.0558*	
H30	1.0444	0.0912	0.3565	0.0720*	
H31	1.2438	0.1947	0.3494	0.0683*	
H32	1.3937	0.2899	0.4746	0.0648*	
H33	1.3411	0.2862	0.6064	0.0500*	
H35	0.6166	0.3365	0.6573	0.0595*	
H36	0.5209	0.4386	0.5888	0.0793*	
H37	0.6600	0.5815	0.5910	0.0811*	
H38	0.8932	0.6230	0.6611	0.0759*	
H39	0.9906	0.5219	0.7297	0.0548*	
H43	0.4982	0.2537	0.3050	0.0508*	
H44	0.5403	0.3953	0.2703	0.0526*	
H45	0.7422	0.5101	0.3372	0.0528*	
H46	0.9024	0.4830	0.4444	0.0525*	
H47A	0.9898	0.3608	1.1050	0.0725*	
H47B	0.8319	0.3648	1.1088	0.0725*	
H47C	0.8695	0.3127	1.0227	0.0725*	
H48A	0.6139	0.1981	0.4749	0.0659*	
H48B	0.7340	0.1656	0.4297	0.0659*	
H48C	0.5776	0.1414	0.3773	0.0659*	

### Atomic displacement parameters (Å^2^)

**Table:**

	*U*^11^	*U*^22^	*U*^33^	*U*^12^	*U*^13^	*U*^23^
Rh1	0.02388 (12)	0.02470 (11)	0.02260 (12)	0.00461 (9)	0.00514 (9)	0.01014 (9)
Rh2	0.02280 (12)	0.02642 (12)	0.02466 (12)	0.00586 (9)	0.00424 (9)	0.01214 (9)
O1	0.0338 (11)	0.0324 (11)	0.0281 (11)	0.0117 (9)	0.0094 (9)	0.0157 (9)
O2	0.0430 (13)	0.0389 (12)	0.0316 (12)	0.0171 (10)	0.0148 (10)	0.0147 (10)
O3	0.0305 (11)	0.0296 (11)	0.0330 (12)	−0.0005 (9)	−0.0001 (9)	0.0156 (9)
O4	0.0288 (11)	0.0375 (11)	0.0283 (11)	−0.0006 (9)	0.0017 (9)	0.0147 (9)
N1	0.0261 (13)	0.0286 (13)	0.0290 (13)	0.0050 (11)	0.0022 (10)	0.0097 (10)
N2	0.0226 (12)	0.0287 (12)	0.0306 (13)	−0.0003 (10)	0.0042 (10)	0.0110 (10)
N3	0.0394 (15)	0.0315 (13)	0.0251 (13)	0.0047 (12)	0.0067 (12)	0.0113 (11)
N4	0.0265 (12)	0.0296 (13)	0.0296 (13)	0.0077 (10)	0.0081 (10)	0.0126 (10)
N5	0.0296 (13)	0.0344 (14)	0.0362 (14)	0.0134 (11)	0.0086 (11)	0.0132 (11)
N6	0.0348 (14)	0.0359 (14)	0.0331 (14)	0.0079 (12)	0.0031 (12)	0.0152 (12)
C1	0.0272 (15)	0.0302 (15)	0.0329 (16)	0.0033 (13)	0.0034 (13)	0.0112 (13)
C2	0.0391 (19)	0.045 (2)	0.056 (3)	−0.0092 (16)	−0.0074 (17)	0.0249 (17)
C3	0.0265 (15)	0.0308 (15)	0.0343 (17)	0.0057 (13)	0.0058 (13)	0.0098 (13)
C4	0.0360 (18)	0.048 (2)	0.0395 (19)	−0.0055 (16)	−0.0019 (15)	0.0109 (16)
C5	0.0257 (15)	0.0302 (15)	0.0287 (15)	0.0045 (12)	0.0042 (12)	0.0111 (12)
C6	0.0447 (19)	0.0475 (19)	0.0424 (19)	0.0237 (16)	0.0186 (16)	0.0196 (16)
C7	0.0357 (17)	0.0343 (16)	0.0383 (18)	0.0125 (14)	0.0106 (14)	0.0106 (14)
C8	0.073 (3)	0.062 (3)	0.057 (3)	0.040 (3)	0.034 (2)	0.021 (2)
C9	0.0297 (16)	0.0262 (14)	0.0303 (16)	0.0015 (12)	−0.0026 (13)	0.0096 (12)
C10	0.045 (2)	0.055 (3)	0.0395 (19)	0.0221 (18)	−0.0025 (16)	0.0090 (16)
C11	0.061 (3)	0.059 (3)	0.056 (3)	0.031 (2)	−0.017 (2)	0.008 (2)
C12	0.073 (3)	0.044 (2)	0.044 (3)	0.003 (2)	−0.026 (2)	0.0112 (17)
C13	0.067 (3)	0.052 (3)	0.0276 (18)	0.001 (2)	−0.0017 (17)	0.0054 (16)
C14	0.0404 (19)	0.0425 (18)	0.0333 (18)	0.0035 (15)	0.0006 (15)	0.0053 (14)
C15	0.0297 (16)	0.0293 (15)	0.0289 (16)	−0.0021 (13)	0.0052 (13)	0.0072 (12)
C16	0.0390 (18)	0.0354 (17)	0.0372 (18)	0.0053 (14)	0.0096 (15)	0.0114 (14)
C17	0.063 (3)	0.0389 (19)	0.050 (3)	0.0017 (18)	0.0063 (19)	0.0211 (17)
C18	0.069 (3)	0.057 (3)	0.057 (3)	−0.011 (2)	0.027 (3)	0.024 (2)
C19	0.054 (3)	0.061 (3)	0.079 (3)	0.002 (2)	0.041 (3)	0.020 (3)
C20	0.0396 (19)	0.0415 (19)	0.061 (3)	0.0080 (16)	0.0222 (17)	0.0181 (17)
C21	0.0369 (17)	0.0289 (15)	0.0302 (16)	0.0079 (13)	0.0031 (14)	0.0103 (13)
C22	0.0363 (17)	0.0421 (18)	0.0296 (16)	0.0099 (14)	0.0099 (14)	0.0158 (14)
C23	0.051 (2)	0.053 (2)	0.049 (2)	0.0242 (18)	0.0218 (17)	0.0284 (17)
C24	0.060 (3)	0.074 (3)	0.058 (3)	0.026 (3)	0.030 (2)	0.045 (3)
C25	0.070 (3)	0.090 (3)	0.049 (3)	0.031 (3)	0.036 (2)	0.039 (3)
C26	0.064 (3)	0.068 (3)	0.043 (2)	0.027 (2)	0.0275 (19)	0.0148 (19)
C27	0.0416 (19)	0.052 (2)	0.0359 (18)	0.0110 (16)	0.0101 (15)	0.0145 (16)
C28	0.0348 (16)	0.0298 (15)	0.0312 (16)	0.0132 (13)	0.0128 (13)	0.0133 (13)
C29	0.044 (2)	0.059 (3)	0.0343 (18)	0.0005 (17)	0.0124 (16)	0.0123 (16)
C30	0.067 (3)	0.078 (3)	0.0289 (19)	0.005 (3)	0.0104 (18)	0.0109 (18)
C31	0.083 (3)	0.063 (3)	0.040 (2)	0.022 (3)	0.035 (3)	0.0233 (19)
C32	0.068 (3)	0.044 (2)	0.057 (3)	0.0045 (19)	0.035 (2)	0.0187 (18)
C33	0.0440 (19)	0.0408 (18)	0.0388 (19)	0.0018 (15)	0.0141 (16)	0.0098 (15)
C34	0.0386 (18)	0.0362 (17)	0.0367 (17)	0.0155 (14)	0.0115 (14)	0.0149 (14)
C35	0.040 (2)	0.044 (2)	0.065 (3)	0.0120 (16)	0.0015 (18)	0.0208 (18)
C36	0.057 (3)	0.068 (3)	0.077 (3)	0.032 (3)	−0.004 (3)	0.025 (3)
C37	0.079 (3)	0.068 (3)	0.078 (3)	0.046 (3)	0.018 (3)	0.039 (3)
C38	0.070 (3)	0.044 (3)	0.094 (4)	0.023 (2)	0.029 (3)	0.037 (3)
C39	0.043 (2)	0.0364 (18)	0.061 (3)	0.0104 (16)	0.0105 (17)	0.0180 (17)
C40	0.0339 (17)	0.0320 (16)	0.0371 (17)	0.0059 (13)	0.0077 (14)	0.0164 (14)
C41	0.0354 (17)	0.0362 (16)	0.0269 (15)	0.0103 (14)	0.0038 (13)	0.0144 (13)
C42	0.0350 (17)	0.0349 (16)	0.0315 (16)	0.0075 (14)	0.0085 (14)	0.0094 (13)
C43	0.0298 (17)	0.054 (2)	0.0344 (18)	0.0025 (16)	−0.0016 (14)	0.0052 (16)
C44	0.044 (2)	0.060 (3)	0.0316 (18)	0.0197 (18)	0.0023 (15)	0.0190 (16)
C45	0.049 (2)	0.0455 (19)	0.042 (2)	0.0106 (17)	0.0011 (16)	0.0253 (16)
C46	0.0404 (19)	0.0428 (19)	0.043 (2)	−0.0043 (16)	−0.0069 (16)	0.0217 (16)
C47	0.085 (3)	0.046 (3)	0.059 (3)	0.019 (3)	0.033 (3)	0.0163 (19)
C48	0.060 (3)	0.0374 (19)	0.064 (3)	0.0005 (18)	0.008 (2)	0.0190 (18)

### Geometric parameters (Å, º)

**Table:**

Rh1—Rh2	2.4241 (4)	C36—C37	1.370 (7)
Rh1—O1	2.034 (2)	C37—C38	1.367 (6)
Rh1—O2	2.028 (3)	C38—C39	1.386 (7)
Rh1—N1	2.061 (2)	C40—C41	1.437 (5)
Rh1—N2	2.071 (2)	C41—C42	1.390 (4)
Rh1—N3	2.236 (3)	C41—C46	1.393 (5)
Rh2—O3	2.0358 (17)	C42—C43	1.399 (5)
Rh2—O4	2.0279 (17)	C42—C48	1.507 (5)
Rh2—N4	2.048 (3)	C43—C44	1.375 (6)
Rh2—N5	2.067 (3)	C44—C45	1.367 (5)
Rh2—N6	2.254 (3)	C45—C46	1.381 (5)
O1—C5	1.282 (4)	C2—H2A	0.960
O2—C7	1.285 (5)	C2—H2B	0.960
O3—C1	1.278 (4)	C2—H2C	0.960
O4—C3	1.281 (4)	C4—H4A	0.960
N1—C1	1.313 (5)	C4—H4B	0.960
N1—C9	1.421 (4)	C4—H4C	0.960
N2—C3	1.311 (4)	C6—H6A	0.960
N2—C15	1.422 (4)	C6—H6B	0.960
N3—C21	1.133 (5)	C6—H6C	0.960
N4—C5	1.309 (5)	C8—H8A	0.960
N4—C28	1.427 (4)	C8—H8B	0.960
N5—C7	1.302 (5)	C8—H8C	0.960
N5—C34	1.431 (5)	C10—H10	0.930
N6—C40	1.137 (5)	C11—H11	0.930
C1—C2	1.506 (4)	C12—H12	0.930
C3—C4	1.506 (4)	C13—H13	0.930
C5—C6	1.506 (5)	C14—H14	0.930
C7—C8	1.512 (6)	C16—H16	0.930
C9—C10	1.374 (5)	C17—H17	0.930
C9—C14	1.384 (5)	C18—H18	0.930
C10—C11	1.388 (6)	C19—H19	0.930
C11—C12	1.372 (6)	C20—H20	0.930
C12—C13	1.368 (7)	C23—H23	0.930
C13—C14	1.382 (5)	C24—H24	0.930
C15—C16	1.385 (5)	C25—H25	0.930
C15—C20	1.386 (5)	C26—H26	0.930
C16—C17	1.391 (6)	C29—H29	0.930
C17—C18	1.372 (7)	C30—H30	0.930
C18—C19	1.370 (7)	C31—H31	0.930
C19—C20	1.385 (6)	C32—H32	0.930
C21—C22	1.449 (5)	C33—H33	0.930
C22—C23	1.383 (6)	C35—H35	0.930
C22—C27	1.395 (5)	C36—H36	0.930
C23—C24	1.381 (6)	C37—H37	0.930
C24—C25	1.369 (6)	C38—H38	0.930
C25—C26	1.371 (7)	C39—H39	0.930
C26—C27	1.388 (6)	C43—H43	0.930
C27—C47	1.506 (6)	C44—H44	0.930
C28—C29	1.380 (4)	C45—H45	0.930
C28—C33	1.375 (5)	C46—H46	0.930
C29—C30	1.380 (6)	C47—H47A)	0.960
C30—C31	1.372 (6)	C47—H47B)	0.960
C31—C32	1.369 (5)	C47—H47C)	0.960
C32—C33	1.383 (6)	C48—H48A)	0.960
C34—C35	1.382 (5)	C48—H48B)	0.960
C34—C39	1.378 (5)	C48—H48C)	0.960
C35—C36	1.388 (7)		
			
Rh2—Rh1—O1	90.04 (7)	N6—C40—C41	176.2 (4)
Rh2—Rh1—O2	89.97 (7)	C40—C41—C42	119.2 (3)
Rh2—Rh1—N1	85.65 (8)	C40—C41—C46	119.6 (3)
Rh2—Rh1—N2	85.90 (8)	C42—C41—C46	121.1 (3)
Rh2—Rh1—N3	172.79 (7)	C41—C42—C43	117.1 (3)
O1—Rh1—O2	179.24 (7)	C41—C42—C48	121.2 (3)
O1—Rh1—N1	88.29 (9)	C43—C42—C48	121.7 (3)
O1—Rh1—N2	91.45 (9)	C42—C43—C44	121.1 (3)
O1—Rh1—N3	97.10 (10)	C43—C44—C45	121.5 (4)
O2—Rh1—N1	90.95 (9)	C44—C45—C46	118.6 (4)
O2—Rh1—N2	89.31 (9)	C41—C46—C45	120.5 (3)
O2—Rh1—N3	82.90 (10)	C1—C2—H2A	109.467
N1—Rh1—N2	171.55 (11)	C1—C2—H2B	109.466
N1—Rh1—N3	95.47 (10)	C1—C2—H2C	109.467
N2—Rh1—N3	92.95 (10)	H2A—C2—H2B	109.477
Rh1—Rh2—O3	89.96 (7)	H2A—C2—H2C	109.476
Rh1—Rh2—O4	89.82 (7)	H2B—C2—H2C	109.474
Rh1—Rh2—N4	85.66 (8)	C3—C4—H4A	109.475
Rh1—Rh2—N5	85.77 (8)	C3—C4—H4B	109.474
Rh1—Rh2—N6	174.59 (6)	C3—C4—H4C	109.471
O3—Rh2—O4	179.63 (9)	H4A—C4—H4B	109.473
O3—Rh2—N4	90.99 (8)	H4A—C4—H4C	109.465
O3—Rh2—N5	88.95 (9)	H4B—C4—H4C	109.470
O3—Rh2—N6	95.24 (9)	C5—C6—H6A	109.474
O4—Rh2—N4	88.69 (8)	C5—C6—H6B	109.472
O4—Rh2—N5	91.33 (9)	C5—C6—H6C	109.483
O4—Rh2—N6	84.98 (9)	H6A—C6—H6B	109.460
N4—Rh2—N5	171.43 (11)	H6A—C6—H6C	109.466
N4—Rh2—N6	95.71 (10)	H6B—C6—H6C	109.473
N5—Rh2—N6	92.83 (11)	C7—C8—H8A	109.470
Rh1—O1—C5	118.8 (2)	C7—C8—H8B	109.460
Rh1—O2—C7	119.3 (2)	C7—C8—H8C	109.470
Rh2—O3—C1	118.88 (19)	H8A—C8—H8B	109.476
Rh2—O4—C3	119.30 (16)	H8A—C8—H8C	109.473
Rh1—N1—C1	121.43 (19)	H8B—C8—H8C	109.479
Rh1—N1—C9	118.18 (19)	C9—C10—H10	119.463
C1—N1—C9	120.3 (3)	C11—C10—H10	119.453
Rh1—N2—C3	120.6 (2)	C10—C11—H11	120.269
Rh1—N2—C15	119.70 (17)	C12—C11—H11	120.282
C3—N2—C15	119.6 (3)	C11—C12—H12	119.998
Rh1—N3—C21	151.6 (3)	C13—C12—H12	119.996
Rh2—N4—C5	122.2 (2)	C12—C13—H13	119.700
Rh2—N4—C28	117.2 (2)	C14—C13—H13	119.711
C5—N4—C28	120.4 (3)	C9—C14—H14	119.968
Rh2—N5—C7	121.3 (3)	C13—C14—H14	119.967
Rh2—N5—C34	119.1 (2)	C15—C16—H16	119.844
C7—N5—C34	119.4 (3)	C17—C16—H16	119.849
Rh2—N6—C40	152.5 (3)	C16—C17—H17	119.958
O3—C1—N1	123.2 (3)	C18—C17—H17	119.962
O3—C1—C2	114.5 (3)	C17—C18—H18	119.987
N1—C1—C2	122.3 (3)	C19—C18—H18	119.982
O4—C3—N2	123.5 (3)	C18—C19—H19	119.851
O4—C3—C4	113.5 (3)	C20—C19—H19	119.860
N2—C3—C4	123.0 (3)	C15—C20—H20	119.771
O1—C5—N4	122.9 (3)	C19—C20—H20	119.778
O1—C5—C6	114.8 (3)	C22—C23—H23	120.097
N4—C5—C6	122.3 (3)	C24—C23—H23	120.111
O2—C7—N5	123.3 (3)	C23—C24—H24	120.451
O2—C7—C8	113.5 (3)	C25—C24—H24	120.448
N5—C7—C8	123.3 (4)	C24—C25—H25	119.408
N1—C9—C10	120.7 (3)	C26—C25—H25	119.392
N1—C9—C14	120.4 (3)	C25—C26—H26	119.366
C10—C9—C14	118.8 (3)	C27—C26—H26	119.365
C9—C10—C11	121.1 (4)	C28—C29—H29	119.723
C10—C11—C12	119.4 (4)	C30—C29—H29	119.726
C11—C12—C13	120.0 (4)	C29—C30—H30	119.850
C12—C13—C14	120.6 (4)	C31—C30—H30	119.847
C9—C14—C13	120.1 (4)	C30—C31—H31	120.316
N2—C15—C16	120.8 (3)	C32—C31—H31	120.323
N2—C15—C20	120.3 (3)	C31—C32—H32	119.731
C16—C15—C20	118.8 (4)	C33—C32—H32	119.732
C15—C16—C17	120.3 (4)	C28—C33—H33	119.815
C16—C17—C18	120.1 (4)	C32—C33—H33	119.812
C17—C18—C19	120.0 (5)	C34—C35—H35	119.887
C18—C19—C20	120.3 (4)	C36—C35—H35	119.885
C15—C20—C19	120.5 (4)	C35—C36—H36	120.023
N3—C21—C22	179.3 (4)	C37—C36—H36	120.014
C21—C22—C23	119.1 (3)	C36—C37—H37	120.040
C21—C22—C27	119.3 (4)	C38—C37—H37	120.045
C23—C22—C27	121.6 (4)	C37—C38—H38	119.666
C22—C23—C24	119.8 (4)	C39—C38—H38	119.677
C23—C24—C25	119.1 (5)	C34—C39—H39	120.079
C24—C25—C26	121.2 (4)	C38—C39—H39	120.074
C25—C26—C27	121.3 (4)	C42—C43—H43	119.449
C22—C27—C26	117.0 (4)	C44—C43—H43	119.445
C22—C27—C47	122.0 (4)	C43—C44—H44	119.247
C26—C27—C47	121.0 (4)	C45—C44—H44	119.248
N4—C28—C29	119.8 (3)	C44—C45—H45	120.673
N4—C28—C33	121.3 (3)	C46—C45—H45	120.682
C29—C28—C33	118.9 (3)	C41—C46—H46	119.747
C28—C29—C30	120.6 (4)	C45—C46—H46	119.754
C29—C30—C31	120.3 (3)	C27—C47—H47A	109.471
C30—C31—C32	119.4 (4)	C27—C47—H47B	109.475
C31—C32—C33	120.5 (4)	C27—C47—H47C	109.464
C28—C33—C32	120.4 (3)	H47A—C47—H47B	109.476
N5—C34—C35	120.4 (3)	H47A—C47—H47C	109.473
N5—C34—C39	120.2 (3)	H47B—C47—H47C	109.468
C35—C34—C39	119.4 (4)	C42—C48—H48A	109.478
C34—C35—C36	120.2 (4)	C42—C48—H48B	109.469
C35—C36—C37	120.0 (4)	C42—C48—H48C	109.476
C36—C37—C38	119.9 (5)	H48A—C48—H48B	109.465
C37—C38—C39	120.7 (4)	H48A—C48—H48C	109.473
C34—C39—C38	119.8 (4)	H48B—C48—H48C	109.466
			
Rh2—Rh1—O1—C5	5.81 (11)	Rh1—N1—C9—C14	−81.3 (3)
O1—Rh1—Rh2—O3	−95.07 (5)	C1—N1—C9—C10	−81.4 (4)
O1—Rh1—Rh2—O4	84.63 (5)	C1—N1—C9—C14	101.2 (4)
O1—Rh1—Rh2—N4	−4.07 (5)	C9—N1—C1—O3	174.2 (3)
O1—Rh1—Rh2—N5	175.98 (5)	C9—N1—C1—C2	−6.6 (5)
Rh2—Rh1—O2—C7	5.09 (13)	Rh1—N2—C3—O4	−4.4 (4)
O2—Rh1—Rh2—O3	84.17 (6)	Rh1—N2—C3—C4	174.48 (17)
O2—Rh1—Rh2—O4	−96.13 (6)	Rh1—N2—C15—C16	−75.3 (3)
O2—Rh1—Rh2—N4	175.16 (5)	Rh1—N2—C15—C20	100.8 (3)
O2—Rh1—Rh2—N5	−4.79 (5)	C3—N2—C15—C16	107.4 (3)
Rh2—Rh1—N1—C1	7.59 (17)	C3—N2—C15—C20	−76.5 (4)
Rh2—Rh1—N1—C9	−169.94 (16)	C15—N2—C3—O4	172.9 (3)
N1—Rh1—Rh2—O3	−6.78 (7)	C15—N2—C3—C4	−8.3 (5)
N1—Rh1—Rh2—O4	172.92 (7)	Rh2—N4—C5—O1	−0.5 (3)
N1—Rh1—Rh2—N4	84.21 (7)	Rh2—N4—C5—C6	−179.35 (13)
N1—Rh1—Rh2—N5	−95.74 (7)	Rh2—N4—C28—C29	−86.7 (3)
Rh2—Rh1—N2—C3	8.12 (16)	Rh2—N4—C28—C33	89.7 (3)
Rh2—Rh1—N2—C15	−169.14 (16)	C5—N4—C28—C29	89.0 (3)
N2—Rh1—Rh2—O3	173.48 (7)	C5—N4—C28—C33	−94.6 (3)
N2—Rh1—Rh2—O4	−6.82 (7)	C28—N4—C5—O1	−176.0 (2)
N2—Rh1—Rh2—N4	−95.53 (7)	C28—N4—C5—C6	5.2 (4)
N2—Rh1—Rh2—N5	84.53 (7)	Rh2—N5—C7—O2	−4.3 (4)
O1—Rh1—N1—C1	97.76 (18)	Rh2—N5—C7—C8	175.70 (15)
O1—Rh1—N1—C9	−79.77 (16)	Rh2—N5—C34—C35	100.6 (3)
N1—Rh1—O1—C5	−79.84 (13)	Rh2—N5—C34—C39	−76.5 (3)
O1—Rh1—N2—C3	−81.82 (18)	C7—N5—C34—C35	−83.6 (3)
O1—Rh1—N2—C15	100.92 (17)	C7—N5—C34—C39	99.3 (4)
N2—Rh1—O1—C5	91.71 (13)	C34—N5—C7—O2	180.0 (3)
O1—Rh1—N3—C21	−164.3 (4)	C34—N5—C7—C8	−0.0 (4)
N3—Rh1—O1—C5	−175.14 (12)	N1—C9—C10—C11	−178.4 (3)
O2—Rh1—N1—C1	−82.30 (18)	N1—C9—C14—C13	178.3 (3)
O2—Rh1—N1—C9	100.16 (17)	C10—C9—C14—C13	0.8 (5)
N1—Rh1—O2—C7	90.74 (15)	C14—C9—C10—C11	−1.0 (5)
O2—Rh1—N2—C3	98.14 (18)	C9—C10—C11—C12	1.3 (6)
O2—Rh1—N2—C15	−79.12 (17)	C10—C11—C12—C13	−1.4 (6)
N2—Rh1—O2—C7	−80.81 (15)	C11—C12—C13—C14	1.2 (6)
O2—Rh1—N3—C21	16.4 (4)	C12—C13—C14—C9	−0.9 (5)
N3—Rh1—O2—C7	−173.86 (14)	N2—C15—C16—C17	178.1 (2)
N1—Rh1—N3—C21	106.7 (4)	N2—C15—C20—C19	−178.6 (2)
N3—Rh1—N1—C1	−165.26 (18)	C16—C15—C20—C19	−2.4 (4)
N3—Rh1—N1—C9	17.21 (17)	C20—C15—C16—C17	1.9 (4)
N2—Rh1—N3—C21	−72.5 (4)	C15—C16—C17—C18	−0.7 (4)
N3—Rh1—N2—C3	−179.01 (18)	C16—C17—C18—C19	−0.1 (5)
N3—Rh1—N2—C15	3.73 (17)	C17—C18—C19—C20	−0.4 (5)
Rh1—Rh2—O3—C1	8.56 (14)	C18—C19—C20—C15	1.7 (5)
Rh1—Rh2—O4—C3	8.06 (15)	C21—C22—C23—C24	178.7 (3)
Rh1—Rh2—N4—C5	3.89 (13)	C21—C22—C27—C26	−178.8 (3)
Rh1—Rh2—N4—C28	179.46 (12)	C21—C22—C27—C47	1.1 (4)
Rh1—Rh2—N5—C7	6.31 (14)	C23—C22—C27—C26	0.5 (5)
Rh1—Rh2—N5—C34	−177.96 (13)	C23—C22—C27—C47	−179.6 (3)
O3—Rh2—N4—C5	93.78 (15)	C27—C22—C23—C24	−0.6 (5)
O3—Rh2—N4—C28	−90.65 (13)	C22—C23—C24—C25	0.1 (5)
N4—Rh2—O3—C1	−77.10 (16)	C23—C24—C25—C26	0.5 (6)
O3—Rh2—N5—C7	−83.72 (16)	C24—C25—C26—C27	−0.6 (6)
O3—Rh2—N5—C34	92.01 (15)	C25—C26—C27—C22	0.0 (5)
N5—Rh2—O3—C1	94.32 (16)	C25—C26—C27—C47	−179.8 (3)
O3—Rh2—N6—C40	−147.8 (4)	N4—C28—C29—C30	177.5 (3)
N6—Rh2—O3—C1	−172.93 (15)	N4—C28—C33—C32	−177.3 (3)
O4—Rh2—N4—C5	−86.03 (15)	C29—C28—C33—C32	−0.8 (5)
O4—Rh2—N4—C28	89.54 (13)	C33—C28—C29—C30	0.9 (6)
N4—Rh2—O4—C3	93.73 (17)	C28—C29—C30—C31	−1.1 (7)
O4—Rh2—N5—C7	96.04 (16)	C29—C30—C31—C32	1.1 (7)
O4—Rh2—N5—C34	−88.24 (15)	C30—C31—C32—C33	−1.0 (7)
N5—Rh2—O4—C3	−77.70 (17)	C31—C32—C33—C28	0.8 (6)
O4—Rh2—N6—C40	32.5 (4)	N5—C34—C35—C36	−179.3 (3)
N6—Rh2—O4—C3	−170.42 (17)	N5—C34—C39—C38	179.3 (3)
N4—Rh2—N6—C40	120.7 (4)	C35—C34—C39—C38	2.3 (5)
N6—Rh2—N4—C5	−170.86 (14)	C39—C34—C35—C36	−2.2 (5)
N6—Rh2—N4—C28	4.72 (13)	C34—C35—C36—C37	1.0 (6)
N5—Rh2—N6—C40	−58.6 (4)	C35—C36—C37—C38	0.2 (7)
N6—Rh2—N5—C7	−178.92 (15)	C36—C37—C38—C39	−0.1 (7)
N6—Rh2—N5—C34	−3.19 (14)	C37—C38—C39—C34	−1.1 (7)
Rh1—O1—C5—N4	−4.5 (3)	C40—C41—C42—C43	176.4 (3)
Rh1—O1—C5—C6	174.46 (11)	C40—C41—C42—C48	−2.9 (5)
Rh1—O2—C7—N5	−1.6 (4)	C40—C41—C46—C45	−176.6 (3)
Rh1—O2—C7—C8	178.38 (12)	C42—C41—C46—C45	0.8 (5)
Rh2—O3—C1—N1	−5.1 (4)	C46—C41—C42—C43	−1.1 (5)
Rh2—O3—C1—C2	175.66 (14)	C46—C41—C42—C48	179.7 (3)
Rh2—O4—C3—N2	−4.0 (4)	C41—C42—C43—C44	0.3 (5)
Rh2—O4—C3—C4	177.07 (14)	C48—C42—C43—C44	179.6 (3)
Rh1—N1—C1—O3	−3.2 (4)	C42—C43—C44—C45	0.7 (6)
Rh1—N1—C1—C2	175.97 (17)	C43—C44—C45—C46	−1.0 (6)
Rh1—N1—C9—C10	96.2 (3)	C44—C45—C46—C41	0.2 (6)
