# Supplementary figures and images for: Crystal structure of tetra­kis­(μ-N-phenyl­acetamidato)-κ4 N:O;κ4 O:N-bis­[(2-methyl­benzo­nitrile-κN)rhodium(II)](Rh—Rh)
Source: Acta Crystallogr Sect E Struct Rep Online. 2014 Aug 20;70(Pt 9):m333–4. doi: 10.1107/S1600536814017930 (PMC4186189; doi:10.1107/S1600536814017930)

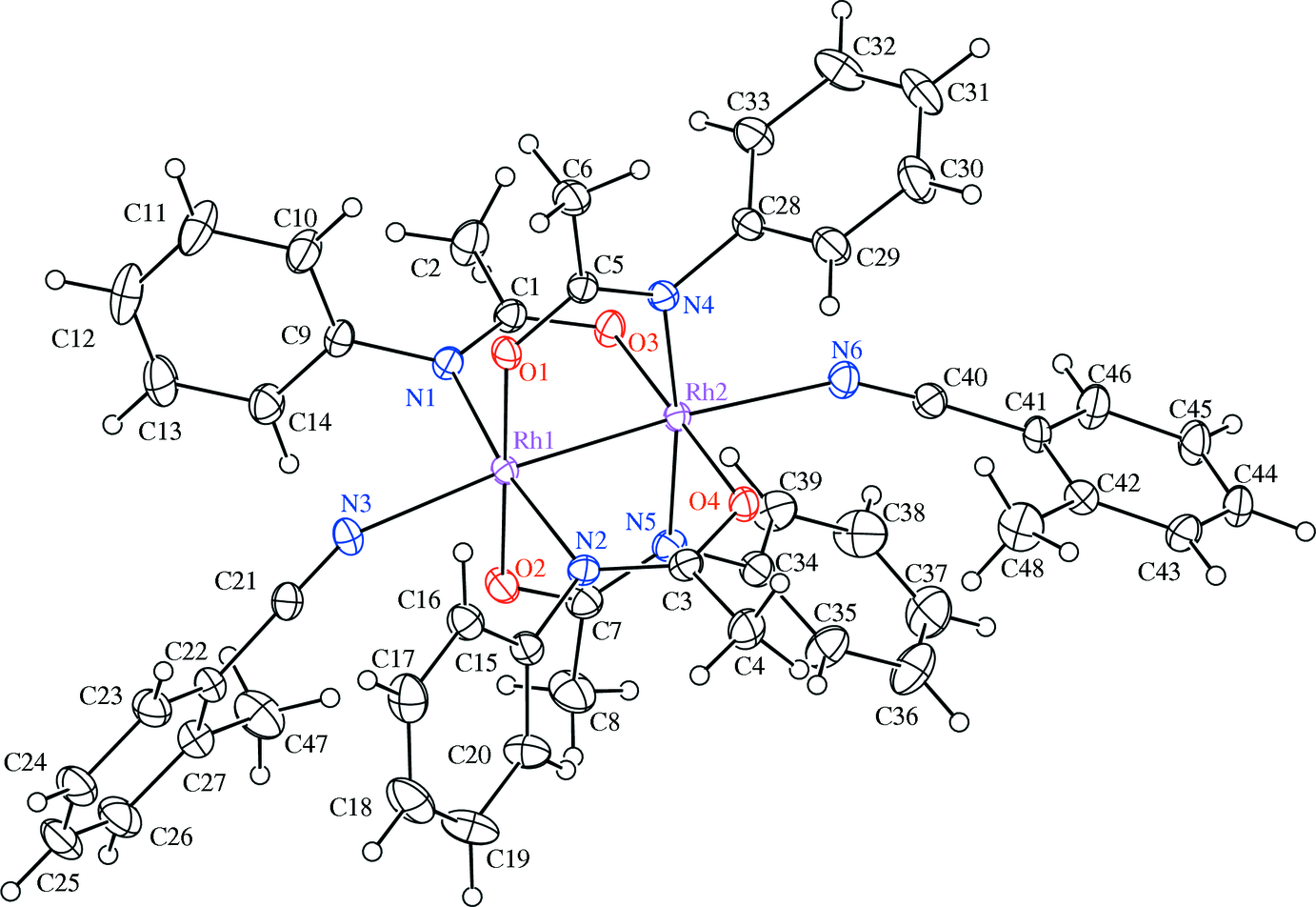

Supplement: Supplementary file 4 [file e-70-0m333-fig1.tif]

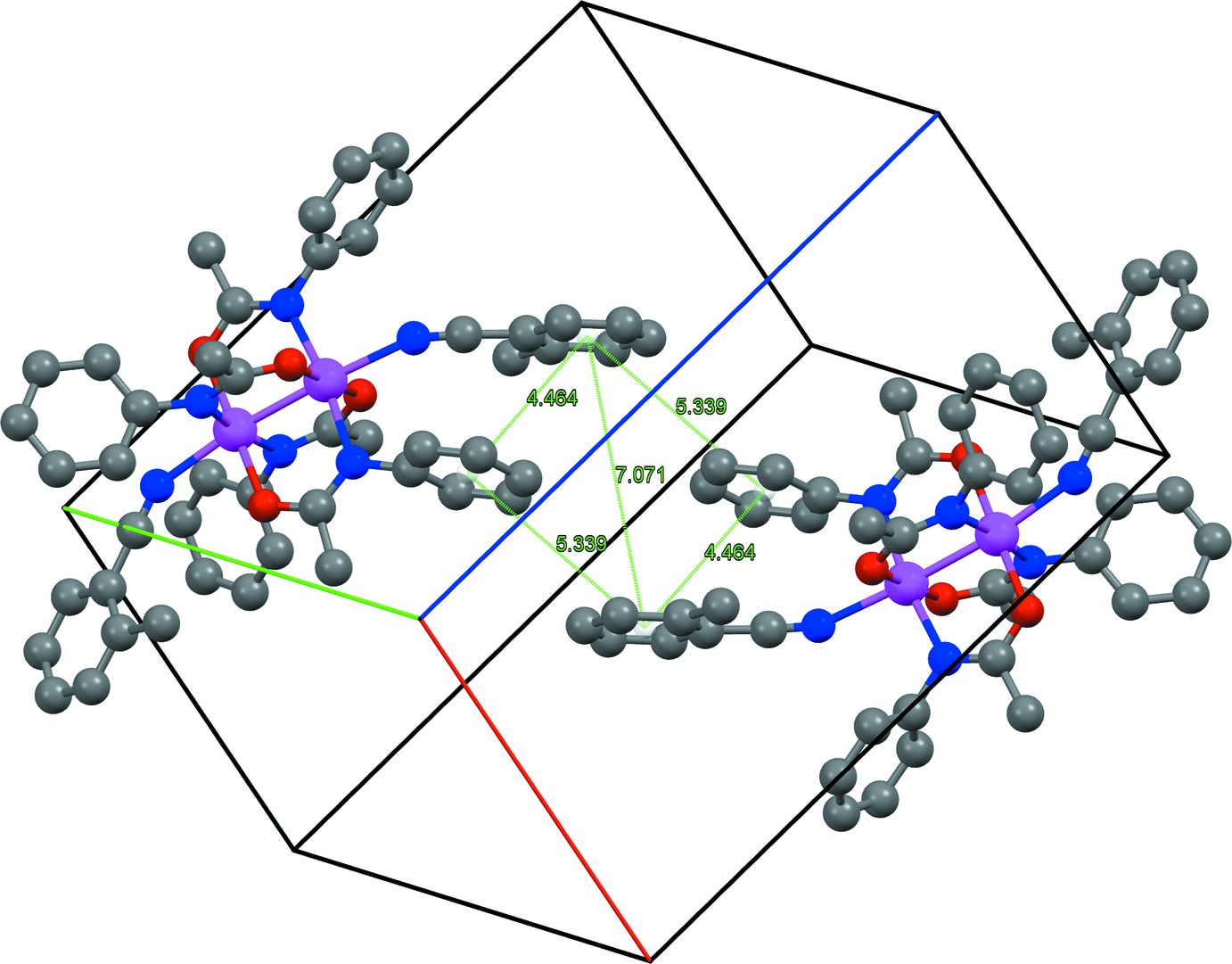

Supplement: Supplementary file 5 [file e-70-0m333-fig2.tif]
